# Brain reward function in people with moderate-to-severe cannabis use disorder who tried to cut down or quit: an fMRI study

**DOI:** 10.1038/s41598-026-50870-y

**Published:** 2026-05-05

**Authors:** Emillie Beyer, Martine Skumlien, Govinda Poudel, Arush Honnedevasthana Arun, Eugene McTavish, Hannah Thomson, Hannah Sehl, Rebecca Segrave, Adam Clemente, Izelle Labuschagne, Peter Rendell, Gill Terrett, Victoria Manning, Chao Suo, Valentina Lorenzetti

**Affiliations:** 1 Neuroscience of Addiction and Mental Health Program, Healthy Brain and Mind Research Centre, School of Behavioural and Health Sciences, Faculty of Health Sciences, Australian Catholic University, Level 5 Daniel Mannix Building, 115 Victoria Parade, Fitzroy, VIC Australia; 2 Department of Social, Genetic & Developmental Psychiatry Centre (SGDP), Institute of Psychiatry, Psychology & Neuroscience, King’s College London, London, UK; 3 Mary MacKillop Institute for Health Research, Australian Catholic University, Melbourne, VIC Australia; 4 Braincast Neurotechnologies, Melbourne, VIC Australia; 5 Centre of Advanced Imaging, Australia Institute for Bioengineering and Nanotechnology (AIBN), University of Queensland, Brisbane, Australia; 6 Urner Institute for Brain and Mental Health, School of Psychological Sciences, Monash University, Clayton, VIC Australia; 7 School of Behavioural & Health Sciences, Australian Catholic University, Fitzroy, VIC Australia; 8 School of Psychology, University of Queensland, Brisbane, QLD Australia; 9 Turning Point, Eastern Health, Monash University, Melbourne, VIC Australia; 10 Clinical Psychopharmacology Unit, Research Department of Clinical, Educational & Health Psychology, University College London, London, United Kingdom; 11 OIMR Berghofer Institute of Medical Research, Queensland Brisbane, Australia

**Keywords:** Diseases, Neuroscience

## Abstract

Cannabis Use Disorder (CUD) affects ~ 50 million individuals globally. CUD has been associated with adverse psychosocial outcomes, including affective flattening, apathy, and anhedonia, which prominent neuroscientific theories ascribe (in part) to altered integrity in brain reward pathways. Yet, emerging evidence from functional neuroimaging (fMRI) is mixed. We examined the activity in key regions-of-interest (ROIs) of the reward neurocircuitry in moderate-to-severe CUD vs. controls, and associations with cannabis use patterns in addition to Apathy Evaluation Scale scores. Brain reward function was examined in 57 people with moderate-to-severe CUD and 35 age and sex-matched controls using the Monetary Incentive Delay fMRI task. We compared brain function between groups, adjusting for age and sex, using exploratory whole-brain and hypothesis-driven ROI analyses in the ventral striatum, dorsal caudate, putamen, insula, cingulate, and orbitofrontal cortices. ROI analyses showed greater activity in the CUD vs. control group in bilateral insula and right putamen when *receiving monetary wins vs. receiving neutral wins* (i.e., outcomes with no monetary gain). The ROI analysis also showed greater activity in the CUD vs. controls in the right putamen when *receiving monetary wins vs. receiving missed monetary wins.* There were no other significant effects for the other ROIs, the whole-brain analyses, or brain-behaviour correlations. Overall, people with CUD showed altered activity in key brain reward regions during the receipt phase of rewards, compared to controls.

**Supplementary Information:**

The online version contains supplementary material available at 10.1038/s41598-026-50870-y.

## Introduction

Cannabis Use Disorder (CUD) is reported in 22% of people who use cannabis^1^, therefore affecting ~ 50 million individuals worldwide^2^. Symptoms of CUD include an inability to control consumption and unsuccessful attempts to cut down or quit using cannabis^3^ despite the experience of adverse outcomes such as mental ill-health (e.g., mood, anxiety, and psychotic disorders^4^); impaired cognition (e.g., learning and memory^5^); and affective flattening (e.g., greater levels of apathy^6^). The global burden of disease for CUD (measured by disability-adjusted life years [DALYs]) increased by 21.2% between 1990 and 2019^7^, reflecting a rising public health concern affecting healthcare (e.g., hospitals and treatment facilities^8^), public safety (e.g., driving while intoxicated and motor vehicle collisions^9^), and economic productivity (e.g., poor work attendance^10^). Therefore, elucidating the mechanisms of CUD is essential to inform the identification of effective treatment targets.

Most neuroscientific theories of addiction suggest the reward system plays a central role, with typically heightened responses to drug rewards and blunted responses to non-drug rewards^11–15^. This notion is confirmed by consistent evidence in Substance Use Disorders other than CUD (SUDs; e.g., alcohol, cocaine, nicotine^16^), and by emerging mixed evidence in cannabis users and CUD. Further, prominent theories, including the incentive-sensitisation theory of addiction^14^, postulate that distinct brain systems underscore distinct phases of reward processing. For example, the ‘anticipation of rewards’ would reflect the incentive motivation (“wanting”) for addictive substances/other rewards, supported by the mesolimbic dopamine system, which can undergo long-lasting sensitisation with chronic consumption^14^. Meanwhile, the ‘receipt of rewards’ would reflect the hedonic impact (“liking”) of consuming substances/other rewards^14^, and in some individuals, it would not change with long-term consumption of substances. Importantly, understanding if CUD is associated with changes in specific reward systems could prove useful to advance a mechanistic understanding of CUD and to inform the development of treatments that target the neurobiology underlying CUD.

Some studies using functional magnetic resonance imaging (fMRI), which measures brain function of cannabis users in vivo, suggest that brain reward function is altered in people who use cannabis compared to controls^6,17^. Specifically, cannabis users have been shown to exhibit altered activity during the *anticipation of monetary rewards* in the nucleus accumbens (NAcc)/ventral striatum^18–21^, a brain area central to reinforcement learning and anticipatory reward processing^22,23^, with a few findings highlighting additional regions (e.g., caudate and whole/dorsal putamen^20,21^). Further, some fMRI studies have found that cannabis vs. control groups show altered brain function during the *receipt of monetary rewards*^6,18^. Yet, findings vary in direction (e.g., greater, lower, and no difference in activity/connectivity) and location of brain functional differences across studies^6,17^. For example, Skumlien et al.^24^ reported greater activity in the inferior parietal cortex and frontal pole in cannabis use vs. controls. Similarly, Van Hall et al.^20^ reported increased activity in the dorsal striatum during the *receipt of monetary rewards* in cannabis users vs. controls. In contrast, other studies have reported no significant group difference during the *receipt of monetary rewards*^25^ or have primarily focused on loss outcomes rather than reward receipt^26,27^.

Preliminary evidence also suggests that brain reward function is associated with cannabis use patterns. For example, the OFC and ACC have been found to correlate with withdrawal symptom scores and the ventral striatum/caudate with cannabis quantity^17^. However, yet again, the direction and significance of the correlations are inconsistent, with both positive, negative, and non-significant correlations^17^. The inconsistent fMRI results on reward processing in cannabis users may be explained in part by methodological limitations. First, the evidence is largely based on samples where CUD status is not confirmed^17,28,29^. Therefore, it is unclear if brain reward function is altered in CUD. Importantly, there are reports that as much as ~ 46% of cannabis users endorse failed attempts to cut down or quit^30^. Thus, it is essential to elucidate the underlying neurobiology in those with unsuccessful quit attempts. Second, only a few studies have examined whether brain reward function in cannabis users is associated with cannabis use patterns^17^ and apathy, which is characterised by reduced motivation and has been ascribed to alterations in the brain’s reward system^31^. Therefore, the role of cannabis use patterns and apathy in the association between cannabis use and brain reward function is unclear. Third, previous fMRI analyses have inconsistently accounted for key demographic variables that affect brain function independently of or in interaction with cannabis use, including age^32^ and sex^33^. As such, age and sex should be accounted for to better elucidate the potential effect of CUD.

This study aimed to investigate how brain reward function is affected during distinct phases of reward processing postulated to be relevant for addiction^14^ – anticipation/’wanting’ and receipt/’liking’ – for the first time in a sample of people with moderate-to-severe CUD who tried to cut down or quit their use in the past two years, compared to controls. In line with the incentive-sensitisation theory of addiction^14^ and emerging fMRI evidence on reward processing in addiction^14^, it was hypothesised that the CUD group vs. controls would show lower activity in the region-of-interest (ROI) ventral striatum, while anticipating rewards. Additionally, we explored if brain activity during both anticipation and receipt phases was altered in other key ROIs implicated in prominent neuroscientific theories of addiction^14^ and with known relevance for reward processing^34,35^: the dorsal striatum (i.e., dorsal caudate and putamen; habit formation), insula (interoception), and the anterior cingulate and orbitofrontal cortices (i.e., inhibitory control and motivation).

Finally, we explored whether brain activity within the CUD group, in the regions that significantly differed between individuals with CUD and controls, was associated with cannabis use patterns (i.e., Cannabis Use Disorder Identification Test [CUDIT], cannabis grams/past month, years of regular use) and levels of apathy, as measured by the Apathy Evaluation Scale (AES).

## Methods

This cross-sectional study was nested within a larger pre-registered project (http://www.isrctn.com/ISRCTN76056942) and received ethical approval from the Australian Catholic University Human Research Ethics Committee (HREC ID: 2019-71H). All methods were carried out in accordance with relevant guidelines and regulations. All participants provided written informed consent before participating in the study.

### Participants

Participants were neurologically healthy adults recruited from the general community in the Melbourne metropolitan area via online advertisements (e.g., Facebook) and local flyers. The CUD and control groups were recruited to be matched on age and sex. Key *inclusion criteria* for the CUD group were: i) used cannabis daily or almost for ≥ 12 months; ii) attempt to quit cannabis use at least once in the last 24 months; iii) endorse DSM-5 criteria for moderate-to-severe CUD, defined as four or more symptoms, confirmed by the Structured Clinical Interview for DSM-5 (SCID-5-RV^36^). Key *exclusion criteria* for all participants included i) illicit substance use (other than tobacco/alcohol) above recreational levels (i.e., 50-lifetime episodes or > weekly use over a 3-month period, other than cannabis in the CUD group); and ii) significant alcohol use defined as scoring ≥ 13 on the Alcohol Use Identification Test scores (AUDIT;^37^. A detailed description of the study’s inclusion and exclusion criteria, recruitment procedure, and testing measures is outlined in Supplementary S1.1—S1.3.

### Face-to-face testing procedure

Participants’ face-to-face assessment for socio-demographic data, cognition, substance use, and mental health lasted ~5 hours with breaks being provided throughout (Supplementary S1.3). Assessments were conducted at the Monash Biomedical Imaging facility in Clayton, Victoria, Australia. All participants were required to provide a urine sample to confirm the level of cannabis metabolites, specifically 11-nor-delta-9-tetrahydrocannabinol-9-carboxylic acid: creatinine (THC-COOH: creatinine), to corroborate self-reported cannabis use in the CUD group and to confirm the lack of cannabis use in controls. Testing started with a study description and written informed consent. Upon testing completion, controls and CUD participants were reimbursed A$100 and A$150 vouchers, respectively, and given a 2D image of their brain. A full description of the face-to-face testing procedure and testing measures is outlined in Supplementary S1.3–1.4

### Imaging methods

All participants were scanned with a 3 T Skyra MRI at Monash Biomedical Imaging in Melbourne between 2019 and 2022. The MRI data acquisition parameters are given in Supplementary S1.5.

#### Monetary incentive delay (MID) fMRI task

The MID fMRI task was administered using a modified version of the original^38^ to analyse changes while anticipating and receiving rewards separately^34^. The instructions and figure of the schematic representation of the MID are located in Supplementary S1.6, Figure S1.

#### fMRI task design

The fMRI task ran for 11 min and 54 s. Participants underwent 20 practice trials to confirm their understanding of the task and to adjust the task to each participant’s mean reaction times (RTs). This system allowed participants to press the button fast enough to win approximately 50% of the trials, which were presented in randomised order (i.e., 15 reward trials out of 30, same for the neutral trials). During the 30 neutral trials, participants were able to win if they hit the button fast enough, but they did not receive any money. Participants completed 30 reward trials (represented by a smiley face cue) and 30 neutral trials (represented by a neutral face). Each cue was presented for 750 ms. Then, a *star symbol* appeared for a mean of 3,286 ms (range: 779–6,729 ms), with an inter-trial mean duration of 3,535 ms (range: 1029–6,979 ms), indicating the task’s anticipatory phase. Then, participants saw an *exclamation mark,* which indicated that they needed to press a button as quickly as possible. Finally, participants received feedback on their performance on the screen, including whether they won money and their cumulative total. Participants could win a total of A$15. This was virtual money; participants understood they would not receive this sum in actual A$.

### Imaging data processing

The data was pre-processed and quality-checked using fMRI prep https://fmriprep.org/en/stable/^39^. Details of pre-processing are outlined in Supplementary S1.7.1.

#### First-level analysis

We examined three contrasts: i) *anticipation of monetary cues vs. anticipation of neutral cues*, ii) *receipt of monetary wins vs. receipt of neutral wins*, and iii) *receipt of monetary wins vs. receipt of missed wins* (see Supplementary S1.7.2 for other details).

#### Second-level analysis

We investigated whole-brain voxel-wise group differences on the contrasts of interest, using independent sample t-tests, with age and sex as covariates. Multiple comparisons correction was performed using a cluster-level FDR correction *p* < 0.05, with an initial uncorrected *p*-value < 0.001 and *k* > 50^40^.

#### Region of interest

ROI analyses were performed in a priori ROIs selected based on Hoogendam et al.^34^, which include the bilateral ventral striatum, dorsal caudate, putamen, insula, and cingulate and orbitofrontal cortices. ROIs were defined using the Anatomic Automatic Labelling (AAL) atlas^41^ and generated using the WFU PickAtlas Toolbox implemented in SPM (version 12). Specifically, the ventral striatum and dorsal caudate were defined as parts of the caudate nucleus located above and below the Z = 0 mm plane. The orbitofrontal cortex ROI included the orbital sections of both the middle and superior frontal gyri. The cingulate cortex ROI was made up of the anterior and medial parts of the cingulate cortex. All other ROIs matched the anatomical regions defined in the AAL atlas. All ROIs were visualised on representative MNI slices at y = 8 (coronal) and z = -8 (axial).

Activation (beta values) within each ROI were extracted by overlapping the ROI masks with the three contrast-of-interest maps generated at the first-level analysis. We adjusted for age and sex by residualising them from the beta values before the analyses. These beta values were entered into a non-parametric Mann–Whitney U test (additional data handling is provided in Supplementary S1.7.3).

### Statistical analysis of behavioural data

#### Descriptive variables

All non-imaging data used in the manuscript were quality-checked via boxplots to inspect for outliers. Data normality tests were performed via the Shapiro–Wilk test and visual inspections, including histograms and quantile–quantile plots. Chi-square tests were used to compare groups on categorical variables (i.e., sex and handedness). Independent sample *t*-test were used to compare groups on normally distributed quantitative variables (i.e., IQ [WASI-II], education years, Perceived Stress Scale [PSS], MID neutral reaction times). Mann–Whitney U tests were used for non-normally distributed variables (i.e., age, Beck Depression Index—II [BDI-II], Community Assessment of Psychic Experiences [CAPE], State-Trait Anxiety Inventory [STAI], Apahty Evaluation Scale [AES], AUDIT, alcohol days/past month, standard drinks/past month, Fagerström Test for Nicotine Dependence [FTND], and MID smile reaction times).

#### Brain-behaviour correlations

We conducted exploratory Spearman’s rank correlations within the CUD group to examine associations between brain activity in the ROIs that significantly differed in the CUD vs. control group and cannabis use patterns (e.g., CUDIT scores, age onset of regular use, grams/past month, and cannabis withdrawal), apathy scores (AES), alcohol problem severity (AUDIT), nicotine severity (FNTD), and MID smile/neutral reaction times. We adjusted for age and sex by residualising them from the beta values before analyses (see Supplementary S1.8). All statistical analyses for behavioural data were conducted using IBM SPSS Statistics version 30, with significance determined as *p* < 0.05.

## Results

### Sample characteristics

Table 1 summarises sample descriptives. The sample comprised 92 people, with a mean age of 28 years, of which 57 (19 female) individuals endorsed a moderate-to-severe CUD with attempts to cut down or quit their cannabis consumption, and 35 (15 female) were controls.

**Table 1:** Overview of sample characteristics.

Variable		CUD		HC		Group difference	
		*M* (*SD*)	Min–Max	*M* (*SD*)	Min–Max	*Z* /*t*^a^/ χ^b^	*p*
Age, yrs		27.23 (8.19)	18–56	28.51 (9.96)	18–55	− 0.46	.649
N, total [F]		57[19]	–	35[15]	–	0.84^b^	.358
Handedness [Right]		57[42]	–	33[31]	–	0.03^b^	.861
Education, yrs		15.28 (2.88)	10–23	15.85 (3.85)	7–25	− 0.81^a^	.211
IQ (WASI-II)		106.58 (9.71)	90–129	108.68 (13.66)	84–135	− 0.81^a^	.202
Mental health							
Depression (BDI-II)		10.84 (6.78)	2–27	7.11 (8.58)	0–37	− 3.21	< .001**
CAPE	Depressive symptoms	23.17 (8.18)	8–45	20.06 (7.37)	10.47	− 1.88	.060
	Positive psychotic symptoms	38.78 (12.16)	20–76	32.49 (8.04)	22–56	− 2.82	.005*
	Negative symptoms	39.67 (12.27)	21–80	33.20 (11.80)	14–53	− 2.12	.034*
State anxiety (STAI-Y)		32.86 (8.61)	20–60	30.66 (8.39)	20–53	− 1.23	.219
Perceived stress (PSS)		15.96 (7.43)	1–33	13.54 (7.54)	1–29	1.50^a^	.138
Apathy (AES)		32.15 (7.38)	20–51	28.97 (7.71)	18–47	− 1.98	.048*
Substance use							
Alcohol	AUDIT	6.93 (4.55)	0–17	2.89 (2.77)	0–13	− 4.39	< .001**
	Alcohol days/pm (TLFB)	6.02 (6.57)	0–30	2.74 (4.41)	0–25	− 3.05	.002*
	Standard drinks/pm (TLFB)	31.23 (48.57)	0–206	10.03 (17.27)	0–81	− 3.22	< .001**
Nicotine	FTND	1.15 (1.77)	0–6	.00	.00	.00	.00
	Days/pm	13.65 (13.83)	0–30	.00	.00	.00	− .00
	Cigarettes/pm	84.58 (143.29)	0–600	.00	.00	.00	− .00
Cannabis	CUD symptoms, N	7.11 (1.86)	4–11	–	–	–	–
	CUDIT	16.18 (5.14)	7 – 30				
	Days /past month	25.75 (4.98)	14–30	–	–	–	–
	Grams/past month	26.85 (21.71)	1 – 84	–	–	–	–
	Age of first use, years	16.5 (2.92)	13 – 32	–	–	–	–
	Age of regular use, years	18.54 (3.50)	14 – 32	–	–	–	–
	Hours from last use	20.71 (11.92)	12 – 73	–	–	–	–
	Withdrawal (CWS)	33.02 (27.72)	0 – 118	–	–	–	–
	THC-COOH:creatinine ng/mg	216.42 (215.74)	5–909	.00	.00	.00	.00
MID	Smile trials, RT	240.44 (21.10)	191–292	256.43 (47.33)	197–417	− 0.911	.362
	Neutral trials, RT	247.32 (33.81)	184–386	260.18 (31.07)	207–328	− 2.05^a^	.041*

^a^, parametric *t-*test. _b_, nonparametric chi-square test. Z, nonparametric Mann–Whitney *U* test; *M,* mean; *SD,* standard deviation; F = Female; pm, past month; N, number, RT, reaction times; CUD, Cannabis User Disorder; HC, Healthy Controls; WASI-II, Wechsler Abbreviated Scale of Intelligence, 2^nd^ Edition; BDI-II, Beck Depression Index – II; CAPE, Community Assessment of Psychic Experiences; STAI-Y, Spielberger State-Trait Anxiety Index – Y; PSS, Perceived Stress Scale; AUDIT, Alcohol Use Identification Test; Age of Onset and Age of Regular Use was measured by Cannabis Use Intervention [CUI], Cannabis/nicotine/alcohol days/grams per month measured by Timeline Follow-Back [TLFB]; FTND*,* Fagerström Test for Nicotine Dependence, CUD symptoms measured by Structured Clinical Interview for DSM-5 Research Version [SCID-5-RV]; CWS, Cannabis Withdrawal Scale; THC-COOH, 11-nor-delta-9-tetrahydrocannabinol-9-carboxylic acid; ng/mg, nanograms per milligram; MID, Monetary Incentive Delay. For *Z* /*t* ^a^/ χ negative sign signifies CUD > HC, a positive sign signifies CUD < HC. *p < .05. **p < .001.

Groups did not significantly differ in years of education completed, IQ, CAPE depressive symptoms, state anxiety, and perceived stress. Compared to controls, the CUD group endorsed significantly higher levels of positive and negative psychotic symptoms, BDI depressive symptom scores, and AES scores.

### Substance use

Compared to controls, the CUD group had significantly higher drinking levels (i.e., AUDIT, standard drinks and consumption days/past month), and higher nicotine dependence scores. In total, 10 of the 58 individuals with a CUD endorsed nicotine dependence (FTND score ≥ 3). No control participant had consumed nicotine in the past month.

### Cannabis use

A *severe* CUD was endorsed by 43 participants, followed by a *moderate* CUD endorsed by 14 participants. The mean age at which individuals with a CUD reported first trying cannabis was about 17 years old, and the mean age for regularly using cannabis (at least 3 times weekly) was 19 years. All participants reported consuming cannabis via smoking joints or bongs, and only 10 of 57 participants additionally reported occasional use of non-smoked products, including edibles (M = 3.67 days of 30) or vapes (M = 4.33 days of 30). On average, the CUD group reported using almost one gram of cannabis daily to almost daily for an average of 26 of 30 days. Additionally, they abstained from using cannabis on average for 20.7 h before testing and reported low levels of withdrawal. We collected urine specimens from all participants to measure urinary levels of THC metabolites (i.e., 11-nor-delta-9-tetrahydrocannabinol-9-carboxylic acid [THC-COOH]), adjusting for creatinine concentration to confirm current cannabis use status in the CUD group and lack of cannabis use in the control group.

### Group differences in RTs during the MID fMRI task

Groups did not significantly differ in reaction times (RTs) for monetary reward trials during the smiley cues. The CUD group were faster than controls during the neutral cues (see Supplementary Figure S2, Section S2.1).

### Neuroimaging results from whole-brain analyses

There were no significant group differences in brain activity for the voxel-wise whole-brain analysis, for any of the contrasts (Supplementary S2.2).

### Neuroimaging results from ROI analysis

There were significant group differences in selected ROIs for receipt outcomes. See below for significant results; additional results are located in Supplementary Table S4, Section S2.3.1.

#### Anticipating monetary cues vs. neutral cues

There were no significant group differences in ROIs activity while *anticipating monetary cues vs. neutral cues* (see supplementary S2.3.2 and Figure S6).

#### Receipt of monetary wins vs. neutral wins

**Fig. 1:**
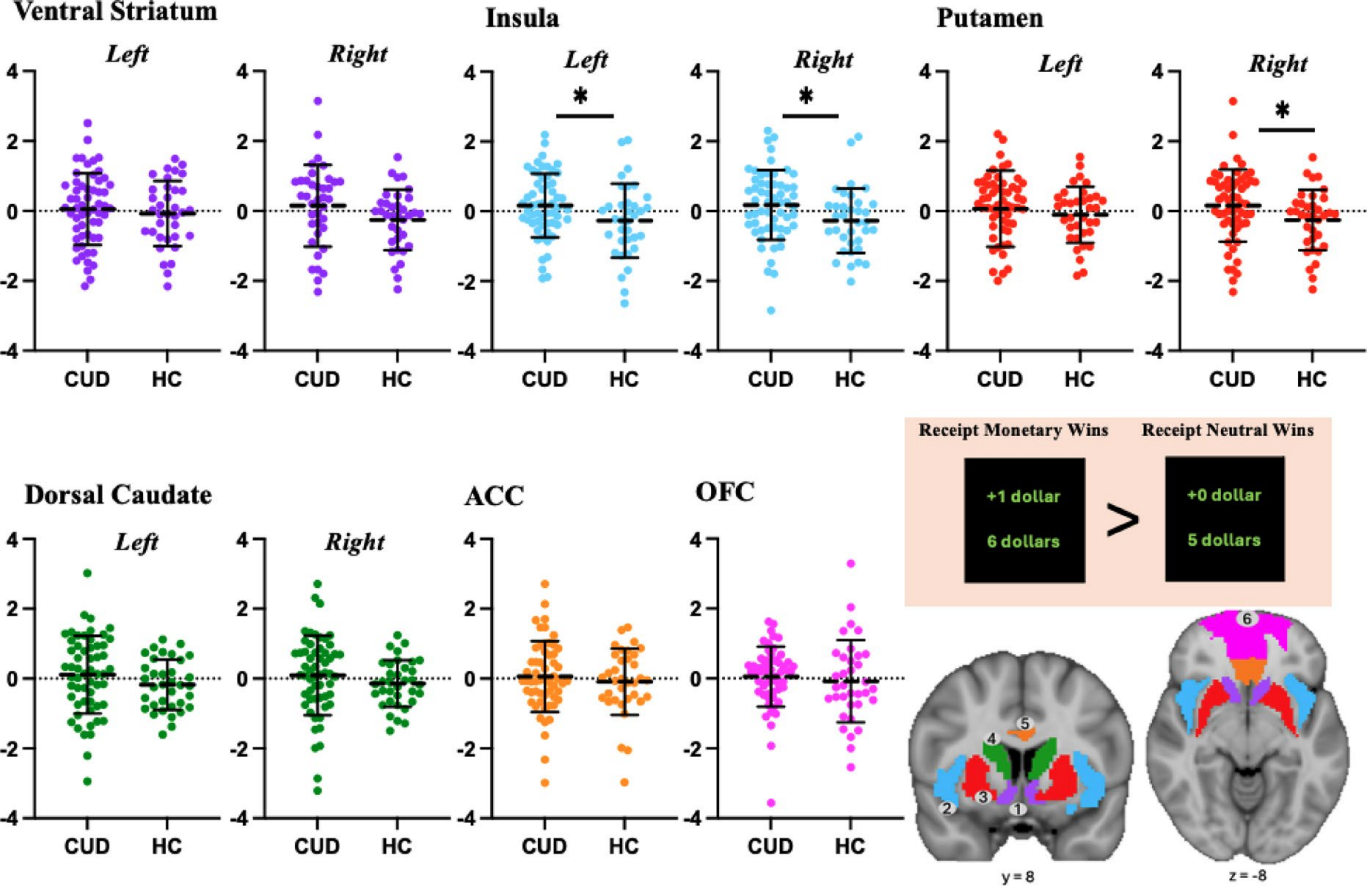
Overview of Results from ROI Analyses, Showing ROI Activity (Beta Values Adjusted for Age and Sex on the Y-axis, Groups on the X-axis: Arbitrary Unit) in CUD vs. Controls while Receiving Monetary Wins > Neutral Wins. *Note*: * = significant; CUD = Cannabis Use Disorder; HC = Healthy Control; ACC = Anterior Cingulate Cortex; OFC = Orbitofrontal Cortex; blacklines represent 95% error bar, indicating a 5% likelihood that the value falls outside the error span; brain image colours signify the selected ROIs: (1) ventral striatum; (2) insula; (3) putamen; (4) dorsal caudate; (5) cingulate cortex; (6) orbitofrontal cortex; adapted from^34^.

Individuals in the CUD group compared to controls, showed greater activity during the *receipt of monetary wins vs. neutral wins* in select ROIs, with moderate effect sizes (Fig. 1): insula (left, *p* < 0.05; *d* = 0.42; right *p* < 0.05; *d* = 0.47) and putamen (right: *p* < 0.05, *d* = 0.43).

#### Receipts of monetary wins vs. missed wins

**Fig. 2:**
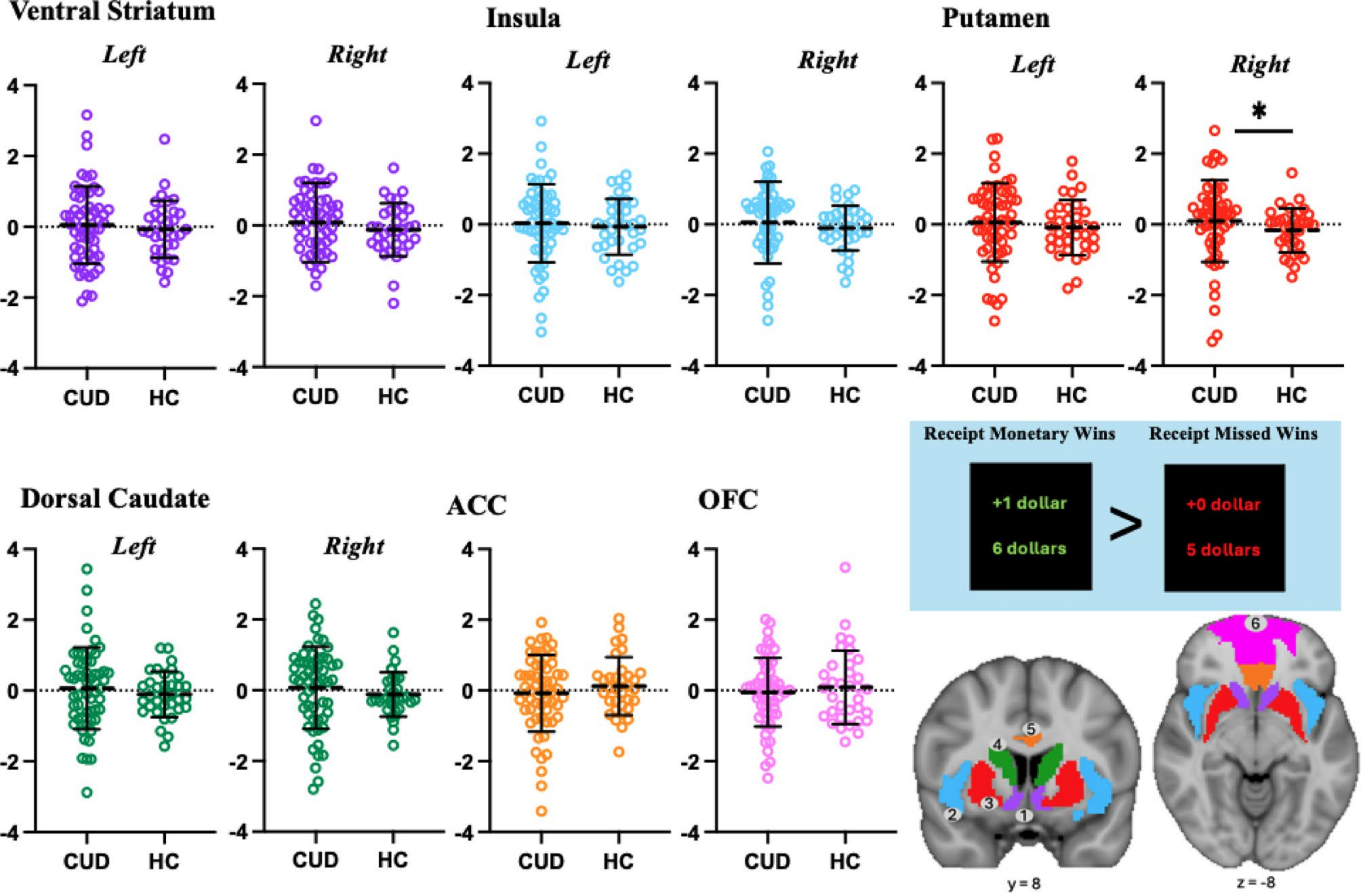
Overview of Results from ROI Analyses, Showing ROI Activity (Beta Values Adjusted for Age and Sex on the Y-axis, Groups on the X-axis: Arbitrary Unit) in CUD vs. while Receiving Monetary Wins vs. Missed Wins. *Note*: * = significant; CUD = Cannabis Use Disorder; HC = Healthy Control; ACC = Anterior Cingulate Cortex; OFC = Orbitofrontal Cortex; blacklines represent 95% error bar, indicating a 5% likelihood that the value falls outside the error span; brain image colours signify the selected ROIs: (1) ventral striatum; (2) insula; (3) putamen; (4) dorsal caudate; (5) cingulate cortex; (6) orbitofrontal cortex; adapted from^34^.

For the contrast *receipt of monetary wins vs. missed wins,* the CUD group showed greater activity than the control group in the putamen (right: *p* < 0.05; *d* = 0.28; small effect size; see Fig. 2).

### Brain-behaviour correlations

No correlations between brain activity and other variables survived correction for multiple comparisons (Bonferroni corrections: 0.05/33 comparisons; adjusted *p* ≤ 0.00152). See Supplementary 2.4, Figure S7 for uncorrected brain-behaviour correlations.

## Discussion

This fMRI study examined brain reward function for the first time in participants with a moderate-to-severe CUD who have attempted to quit or cut down their cannabis use. The individuals with CUD, compared to controls, showed greater activity bilaterally in the insula and right putamen while *receiving monetary wins vs. neutral wins.* The CUD vs. the control group also showed greater right putamen activity while *receiving monetary wins vs. missed wins*. Contrary to our hypothesis, there was no significant group difference while *anticipating monetary cues vs. anticipating neutral cues*. Overall, our findings suggest that alteration of reward processing in CUD may be specific to receiving rewards (and not to anticipating rewards). The findings partly align with prominent neuroscientific theories of addiction that postulate altered activity in habit-related (e.g., dorsal striatum) and interoceptive (e.g., insula) pathways during the ‘liking’/receipt phase of rewards^11,15^; but do not support neuroadaptations during the ‘wanting’ phase of reward processing in this sample.

### Receipt of monetary wins and cannabis use

We found that the CUD vs. control group showed greater activity in the putamen and insula during the ‘liking’ phase of reward processing measured during the *receipt of monetary wins*. Specifically, greater putamen activity was found across both receipt contrasts (*receiving monetary wins vs. neutral wins* and *missed wins*), consistent with a previous study that found increased putamen activity in cannabis users vs. controls while *receiving monetary wins*^20^. Notably, the putamen (part of the dorsal striatum) is implicated in habit formation and automated/compulsive substance use^11,43,44^. Therefore, increased putamen activity during the *receipt of monetary wins* may reflect reinforcement processes that contribute to the development of habitual substance use.

We also found greater insula activity in the CUD group vs. controls during the *receipt* of *monetary wins* vs. the *receipt of neutral wins.* Although some previous studies have reported greater insula activity in cannabis users vs. controls during other reward/loss conditions^21,26^, no study has found a significant group difference in insula activity during the *receipt of monetary wins* (e.g.,^20,24,25^). Interestingly, the insula is implicated in arousal and interoception^45^, and is often altered in SUDs^46^. Therefore, greater insula activity while *receiving monetary rewards* in the CUD group may reflect increased awareness of bodily states or greater salience attribution. However, further research is needed to understand the role of insula activity while *receiving monetary rewards* in people with CUD.

### Anticipation of monetary cues and cannabis use

No significant group differences were found in brain activity during the ‘wanting’ phase of reward processing, measured while *anticipating monetary cues vs. neutral cues.* This aligns with several previous studies^24–27,47^, but needs to be interpreted with caution. Indeed, brain activity was not robust within the control group for this contrast, suggesting that this contrast might not have robustly recruited neural pathways related to anticipation, possibly due to the lack of real monetary rewards.

At the behaviour level, we also found no differences in RTs when responding to monetary cues, but faster RTs towards neutral cues, suggesting that at a behavioural level, there might have been a blunting of anticipation of rewards; and a dissociation between the behavioural and neural correlates of reward anticipation. Our findings, in conjunction with existing evidence, demonstrate inconsistent alterations of the neural correlates of reward anticipation in CUD.

### Strengths and limitations

A main strength of the current study is the novel sample of individuals with a moderate-to-severe CUD who have tried to cut down or quit using cannabis in the past two years. By understanding how those with more severe forms of CUD process rewards, treatment targets can be better informed.

Nevertheless, several important methodological limitations must be considered when interpreting these findings. First, the cross-sectional design limits conclusions about directionality; we cannot determine whether greater putamen and insula activity during the receipt phase of rewards reflects neuroadaptations from chronic cannabis use or pre-existing traits that increase vulnerability to CUD. Future longitudinal studies are needed to track how these patterns change over time and how they relate to changes in cannabis use.

Second, while providing a robust methodology to measure the activity of the brain’s reward neurocircuitry, the controlled fMRI environment, where participants performed the task, and the structure of the MID may not accurately reflect how people process rewards in real-life situations^48^.

Third, the results might reflect, instead of cannabis use-related effects on brain integrity, the effects of nicotine and alcohol use, and related problems, or other measures of hedonic tone (e.g., anhedonia). These factors may influence brain function independently and in interaction with cannabis^16^. Indeed, nicotine was consumed by most participants in the CUD group but by none in the control group; alcohol was consumed in greater amounts and on more days by participants in the CUD group than by controls; and we did not measure anhedonia. Further, we did not measure nicotine withdrawal because we did not ask participants to abstain from nicotine use, and we allowed participants to consume nicotine during scheduled breaks. Yet, we could not confirm that nicotine withdrawal in some participants might have affected brain reward function. Future work is required to unpack the independent and interactive effects of CUD, cannabis, alcohol, and nicotine consumption and related problems, and of distinct metrics of hedonic tone, on the neurocircuitry of reward processing. Fourth, the current study did not examine loss-related contrasts. Previous studies using the MID reported blunted neural responses to loss outcomes in cannabis users^25,27^. As such, future research should incorporate loss-related contrast (e.g., losing monetary rewards) to clarify whether CUD is associated with altered reward processing of negative outcomes.

Fifth, the strict exclusion criteria used during recruitment may have resulted in a sample that is not fully representative of individuals who endorse a CUD. While the exclusion criteria were designed to meet the objectives of the experiments, future research should include cannabis users with polydrug use and comorbid psychiatric disorders to better understand their impact on brain reward function and improve generalisability. Sixth, while the study employed robust metrics of cannabis consumption (e.g., TLFB^49^), reliance on self-report measures introduces the potential for underreporting biases due to recall, stigma, and lack of awareness^50^. Although urinary THC metabolites screening was included, which provides an objective indicator of recent cannabis use, future studies should incorporate international standardised quantification metrics, such as the iCannToolkit^51^, the ‘roll a joint paradigm’^52,53^, and tools that measure the amount of THC in participants’ cannabis products (e.g., Standard THC units^42^) to improve accuracy. Finally, consistent with prior work investigating reward processing using the MID fMRI task^34^, corrections for multiple comparisons were not applied to the exploratory ROI analyses. As such, results should be interpreted with caution.

### Conclusions

We examined brain reward function for the first time in a sample of participants with a moderate-to-severe CUD with previous attempts to quit or cut down their cannabis use. The findings suggest that the CUD group showed greater activity in the putamen and insula during the receipt phase of rewards compared to controls. These current findings are partly in line with prominent neuroscientific theories of addiction that postulate altered activity in key brain reward regions during the receipt phase of monetary reward^11,15^. Future longitudinal neuroimaging studies are required to determine whether changes in brain reward function predate or follow the onset of CUD.

## Supplementary Information

Below is the link to the electronic supplementary material.

Supplementary Material 1

## Data Availability

The datasets generated during and/or analysed during the current study are available from the corresponding author upon reasonable request.
