# Supplementary material for: Brain reward function in people with moderate-to-severe cannabis use disorder who tried to cut down or quit: an fMRI study
Source: Sci Rep. 2026 May 5;16:20716. doi: 10.1038/s41598-026-50870-y (PMC13333791; doi:10.1038/s41598-026-50870-y)
Supplement: Supplementary file 1 — Supplementary Material 1 [file 41598_2026_50870_MOESM1_ESM.docx]

**Supplementary Data**

1. **Supplementary Methods**
   1. **Participants’ Selection Criteria**
      1. **Inclusion Criteria**

*Inclusion criteria for all participants were:* i) age *18-to-55* years; ii) fluency in English; iii) normal to corrected vision; iv) meeting MRI safety criteria; v) able to attend sessions at the Monash Biomedical Imaging facility.

*Inclusion criteria for the Cannabis Use Disorder (CUD) group were:* i) had used cannabis daily-to-almost daily for ≥ 12 months; ii) attempt to quit cannabis use at least once in the last 24 months; iii) endorse DSM-5 criteria for moderate-to-severe CUD, defined as four or more symptoms, confirmed by the Structured Clinical Interview for DSM-5 (SCID-5-RV [1]).

- - 1. **Exclusion Criteria**

*Exclusion criteria for all participants were*: i) significant medical conditions, history of acquired brain injury (e.g., or loss of consciousness > 5 minutes) or neurological disorders (e.g., stroke, epilepsy); ii) any diagnosed psychiatric disorders confirmed by the Mini International Neuropsychiatric Interview [MINI] [2]; except for low-to-moderate depression/anxiety that is highly entrenched with CUD; iii) illicit substance use (other than tobacco/alcohol) above recreational levels (i.e., greater than 50-lifetime episodes, or > weekly use over 3-months (other than cannabis in the CUD group), iv) illicit substance use 4 weeks before testing (other than cannabis in CUD group); v) illicit substance and alcohol use 12-hours prior to testing; vi) significant alcohol use of scores ≥ 13 measured via the Alcohol Use Identification Test scores (AUDIT [3]); vii) use of prescribed medication affecting the central nervous system (except for antidepressant – e.g., selective serotonin reuptake inhibitors); viii) presence of MRI contradictions (e.g., pacemaker) confirmed by a screener from the testing facility; ix) currently breastfeeding or pregnant; and x) intelligence quotient score < 80 as per Wechsler Abbreviated Scale of Intelligence – second edition (WASI-II [4]). *Exclusion criteria for the control group were:* i) no use of cannabis in the 12 months prior to testing.

As a result of the inclusion/exclusion process, 24 participants were excluded due to: i) IQ below 80 (*n* = 1); missing neuroimaging data for the MID (*n* = 7); ii) fMRI head movement issues (*n* = 8); iii) neurological disorders (*n* = 2); iv) incidental finding (*n* = 3); v) control participants’ with recent use substances (*n* = 2), CUD participant with synthetic cannabis use (*n* = 1).

- 1. **Procedure**

Community members interested in completing the study were directed to an online screening questionnaire using Qualtrics version XM. To determine participants’ eligibility against the study’s selection criteria for either the CUD or control group, a detailed phone screening interview was conducted, which included: i) demographics (e.g., age, sex); ii) MRI safety screening; iii) previous research participation in monitoring recent interventions that could affect brain outcomes (e.g., mindfulness); iv) handedness; v) past and current cannabis use; vi) substance use history measuring lifetime, past year, past 6 months and past 3 months of substance use; viii) screening for major mental health disorders (i.e., MINI [2]; ix) depressive symptoms as per the Depression, Anxiety, and Stress Scale (DASS [5], and x) attempts to quit or cut down on cannabis in the past 24 months.

In total, 9,045 people completed the online screening survey. Of this, ~1,228 community members received a phone call from a student researcher to confirm their eligibility. After completing the comprehensive phone screening interview, ~116 individuals were asked to attend a face-to-face testing session at the Monash Biomedical Imaging facility in Clayton, Victoria, Australia.

- 1. **Face-to-Face Testing Procedure**

The cross-sectional assessment comprised one face-to-face testing session, which lasted ~5 hours. Participants received short breaks throughout, and one longer, ~15-minute lunch break with snacks was provided after the MRI scan. The first half of the session included: i) study description and participants providing written informed consent, ii) sociodemographic data collection; iii) semi-structured interviews relating to substance use (i.e., Structured Clinical Interview for DSM-5, Research Version [SCID-5-RV], Marijuana Cravings Questionnaire [MCQ], and mental health measures (i.e., State-Trait Anxiety Inventory [STAI]. Participants were then instructed to have a short break.

The second half of the session consists of: i) MRI safety protocols, and ii) the fMRI task – the MID that measures the dependent variable of the first study (i.e., brain function during the anticipation and receipt phase of reward in CUD vs. controls). After the scan, participants were instructed to have a lunch break. Then they completed structured questionnaires including: i) cognitive tasks (i.e., WASI-II, substance use measures (i.e., 30-day Timeline Follow-Back [TLFB], Marijuana Ladder, Cannabis Withdrawal Scale [CWS], CUDIT-R, AUDIT, Fagerström Test for Nicotine Dependence [FTND], and ii) mental health measures (i.e., Beck Depression Index - II [BDI-II], Community Assessment of Psychic Experiences [CAPE], Perceived Stress Scale [PSS], and Apathy Evaluation Scale [AES]. Control participants then received a debrief and reimbursement of an AUD $100 voucher and could request a 2D image of their brain (the CUD group received reimbursement after completing session 2, which was about ~2 weeks later). For this manuscript, we solely focused on the cross-sectional assessment.

- 1. **Testing Measures**

Participants’ demographic data, including age, biological sex (i.e., male/ female), and years of education, were collected as part of a screening survey. IQ was measured using the WASI-II [4]. Handedness was measured using the Edinburgh Handedness Inventory—Short Form (EHI-SF) [6]. The tools used to characterise samples’ substance use and psychopathology are outlined below.

- - 1. **Cannabis and Substance Use**

The severity of CUD was confirmed via the SCID-5-RV [1]. The onset and duration of regular use (i.e., > 1 time monthly) and the age of the first cannabis try were assessed via a semi-structured interview used by our extended team [7-9]. Cannabis withdrawal was measured with CWS [10]. Participants' days of cannabis, tobacco, alcohol, and other substance use in the past month and quantity of use (e.g., cannabis grams, cigarettes, standard drinks) were measured using the TLFB [11]. Problematic alcohol use was measured using the AUDIT [3], and participants' severity of nicotine dependency was measured using the FTND [12]. Greater scores on these measures signify greater problematic substance use/symptoms.

- - 1. **Psychopathology Symptom Scores**

We measured scores for depression symptoms using the BDI-II [13], as well as psychotic depressive, positive, and negative symptoms using the CAPE [14]. Subjective stress was measured using the PSS [15] and state anxiety via the STAI-Y [16]. Finally, Apathy scores were measured using the AES [17]. Greater scores on these measures indicated greater psychopathology symptoms.

- 1. **Imaging Methods**

All participants were scanned with a 3 T Skyra MRI at Monash Biomedical Imaging in Melbourne between 2019 and 2022. T1-weighted images were acquired using a three-dimension (3D) magnetization-prepared rapid gradient-echo (MP-RAGE), with 192 slices, voxel size = 1×1×1 cubic millimetre (mm^3^), acquisition time = 5:12 min, repetition time = 23 milliseconds (ms), echo time (TE) = 2.07 ms, field of view = 256mm by 256mm, inversion time (TI) = 900 ms, flip angle = 9°. The functional scan applied a gradient-echo-planar sequence with repetition time (TR) = 1600 ms, echo time (TE) = 23 ms, field of view = 190mm by 190mm, flip angle = 72°, in-plane resolution = 3 mm by 3 mm, slice thickness = 3.5 mm and 33 slices.

- 1. **Monetary Incentive Delay (MID) fMRI Task**

Figure S1 presents a schematic representation of the MID. The MID was administered using an adapted version [18, 19]; the instructions included: “*In this task, your goal is to win as much (virtual) money as possible. You will see a smiley face, followed by a star, and then an exclamation mark. Your job is to press the blue button (on the left) as quickly as possible when you see the exclamation mark. Press the button now to practice. There will be a short practice run before the task.”*


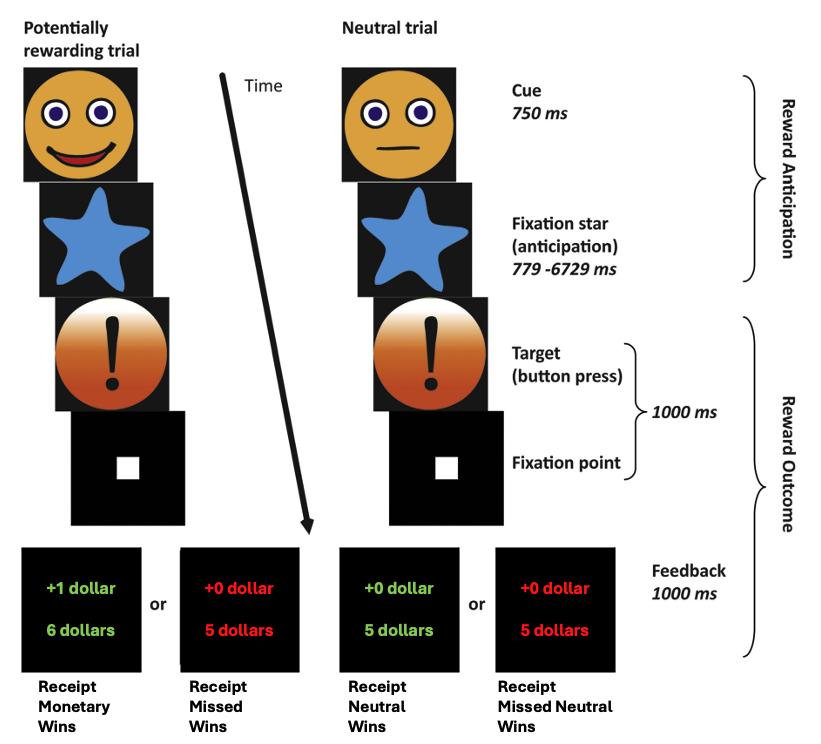
**Figure S1.** *Schematic Representation of the Monetary Incentive Delay (MID) fMRI Task*

Note. Figure S1 is a schematic representation of the MID, adapted from Hoogendam et al. [19]; ms = milliseconds.

- 1. **Imaging Data Processing**
     1. **Data Pre-processing and Quality Checks**

The steps comprised slice time corrections of distortion, co-registration, normalisation to standardising spaces (i.e., Montreal Neurological Institute [MNI] space) and smoothing with a 6mm Gaussian kernel. The quality of images was examined using Framewise Displacement (FD), followed by Derivatives of Root Mean Square Variance over Voxels (DVARS) to gauge noise levels and voxel-wise signal plots (i.e., carpet plots). FD parameters were measured using conservative criteria [20]. In cases where motion surpassed the set criteria (*n* = 8). In total, 15 participants were removed due to the fMRI quality checks.

- - 1. **First Level Analysis**

The first-level analyses were computed using SPM (version 12) via MATLAB (version r7771). The analysis used a general linear model (GLM) to measure the associations between the detected blood-oxygen-level-dependent (BOLD) and six factors indicative of haemodynamic variations: i) anticipating monetary cues; ii) anticipating neutral cues; iii) receipt of monetary wins; iv) receipt of missed monetary wins; v) receipt of neutral wins; vi) receipt of missed neutral wins.

Additionally, six motion estimations, which translate and rotate across the x, y, and z axes, were selected as covariates of no interest in the CUD and control groups. The onset of the anticipation factors was set at the time of cue presentation, with a duration range of 1529–7479 ms. However, we did not vary the cue duration (1000ms) during feedback. We examined three contrasts: i) anticipation of monetary cues vs. anticipation of neutral cues, ii) receipt of monetary wins vs. receipt of neutral wins, and iii) receipt of monetary wins vs. receipt of missed monetary wins.

- - 1. **Additional Data Handling**

We assessed the distribution of beta values for each ROI using boxplots and histograms and the Shapiro-Wilk test for normality in SPSS. Visual inspections and normality tests indicated that the majority of the data was non-normally distributed; therefore, we used Mann-Whitney U tests to compare groups across ROIs. Outliers were identified via box plots, using the standard 1.5 interquartile range rule [21]. Analyses were re-run with and without these outliers; as results remained unchanged, all outliers were retained. For the *Anticipating Monetary Cues vs. Neutral Cues* contrast, outliers were detected in the right insula (*n* = 1), left insula (*n* = 1), left putamen (*n* = 1), and right putamen (*n* = 2). For the *Receipt of Monetary Wins vs. Missed Wins* contrast, one outlier was identified in the orbitofrontal cortex (OFC; *n* = 1). No other outliers were identified.

- 1. **Brain-behaviour Correlations**

A correlation matrix was generated using Spearman’s rank correlation coefficients (rₛ) to investigate the strength of correlation between variables. The following thresholds were used to interpret the strength of the correlation: weak (rₛ = 0.01-0.39), moderate (rₛ = 0.40-0.59), and strong (rₛ ≥ 0.60). Variables of interest were selected based on theoretical relevance, and additional variables were removed to reduce collinearity. Specifically, perceived stress and state anxiety were excluded because they correlated with apathy scores. Alcohol use variables (days/past month and standard drinks/past month) were removed because of their strong correlation with AUDIT scores. Nicotine variables (days/past month and dose/past month) were excluded due to their strong correlation with FNTD. The SCID was excluded due to its correlation with CWS. Cannabis days/past month and hours of last use were removed due to their correlation with cannabis grams/past month. Finally, the age of cannabis onset was excluded due to its strong correlation with the age of regular cannabis use.

1. **Results**
   1. **Group Differences in Monetary Incentive Delay fMRI Task**

Figure S2 outlines the group difference in smile reaction times compared to neutral reaction times during the MID*.* Groups did not significantly differ in reaction times for reward trials (during the smiley cue), but the CUD group showed faster reaction times than controls during neutral trials.

** Figure S2.** *Group Differences between CUD and Control Participants during the MID fMRI Task*

Note. MID = Monetary Incentive Delay Task; ns = non-significant; * = significant; CUD = Cannabis Use Disorder; HC = Healthy Controls; redlines represent 95% error bars, indicating a 5% likelihood that the value falls outside the error span.

- 1. **Neuroimaging Results from Whole-Brain Analysis**
     1. ***Anticipation of Monetary Cues vs. Anticipation of Neutral Cues***

The CUD group, compared to controls, did not significantly differ after multiple comparisons at a whole-brain level while anticipating monetary cues vs. anticipating neutral cues (cluster threshold k >5 voxels, *p* < 0.001; see Figure S3)

**Figure S3.** *Overview of Non-significant Brain Activity in CUD Compared to Controls while Anticipating Monetary Cues > Anticipating Neutral Cues*

**
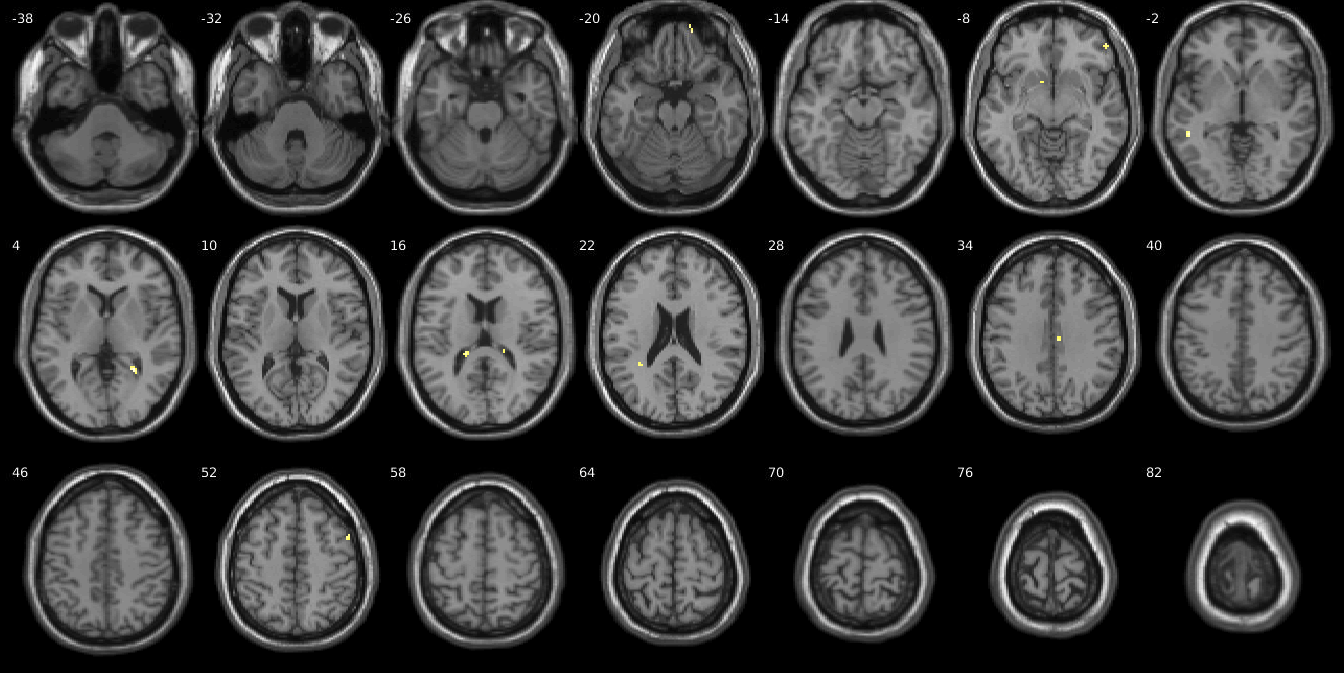
**

Note. Cluster threshold k >5 voxels, *p* < 0.001.

**Table S1.** *Overview of Location and Strength of Peak Clusters Showing Non-significant Brain Activity in CUD compared to Controls while Anticipating Monetary Cues > Neutral Cues*

| Cluster-level | | | MNI Coordinates | | | T score |
| --- | --- | --- | --- | --- | --- | --- |
| *P*  _FWE-corr_ | *k _E_* | *P*  _uncorrected_ | X | Y | Z |  |
| ***anticipating cues > neutral cues*** | | | | | | |
| 0.953 | 22 | 0.047 | 28 | -48 | 4 | 4.16 |
| 0.999 | 13 | 0.116 | 20 | 60 | -18 | 4.09 |
| 1.000 | 6 | 0.276 | 28 | 46 | -18 | 3.93 |
| 0.975 | 20 | 0.056 | -48 | -40 | -2 | 3.92 |
| 1.000 | 7 | 0.240 | 16 | -34 | 16 | 3.74 |
| 1.000 | 9 | 0.185 | -32 | -44 | 20 | 3.64 |
| 1.000 | 8 | 0.210 | 52 | 46 | -8 | 3.61 |
| 0.999 | 13 | 0.116 | 6 | -20 | 32 | 3.61 |
| 1.000 | 9 | 0.185 | 50 | 8 | 52 | 3.53 |
| 1.000 | 5 | 0.320 | -6 | -26 | 18 | 3.50 |
| 1.000 | 6 | 0.276 | -8 | 10 | -10 | 3.47 |
| 1.000 | 5 | 0.320 | 20 | -32 | 18 | 3.45 |

*Note.* All coordinate were presented in Montreal Neurological Institute (MNI) space. Cluster threshold k >5 voxels, *p* < 0.001.

- - 1. ***Receipt of Monetary Wins vs. Receipt of Neutral Wins***

There were no significant group differences after multiple comparisons at a whole-brain level while *receiving monetary wins vs. receiving neutral wins* (cluster threshold k >5 voxels, p < 0.001; see Figure S4).

**Figure S4.** *Overview of Non-significant Brain Activity in CUD Compared to Controls while Receiving Monetary Wins> Receiving Neutral Wins*


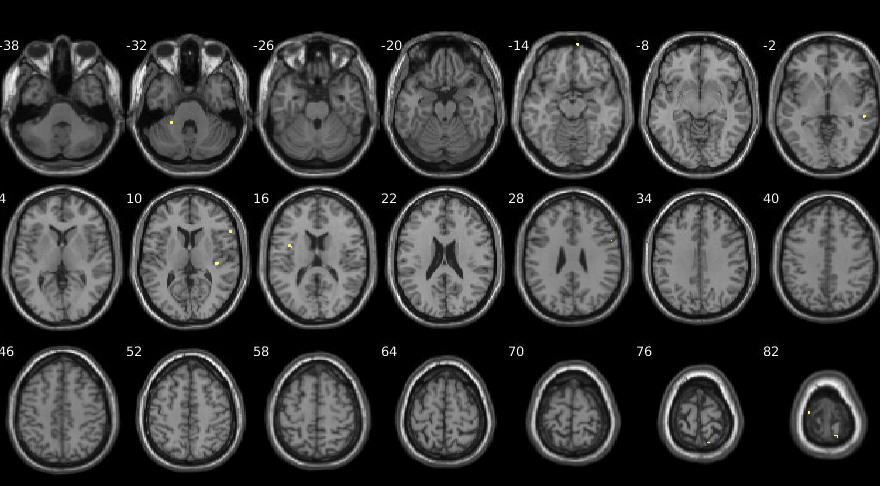


Note. Cluster threshold k >5 voxels, *p* < 0.001.

**Table S2.** *Overview of Location and Strength of Peak Clusters Showing Non-significant differences in Brain reward Activity in CUD compared to Controls while Receiving Monetary Wins > Receiving Neutral Wins*

| Cluster-level | | | MNI Coordinates | | | T score |
| --- | --- | --- | --- | --- | --- | --- |
| *P*  _FWE-corr_ | *k _E_* | *P*  _uncorrected_ | X | Y | Z |  |
| *receipts of wins > neutral wins* | | | | | | |
| 0.993 | 16 | 0.066 | 38 | -22 | 8 | 4.41 |
| 1.000 | 6 | 0.243 | 10 | 72 | -14 | 4.23 |
| 0.971 | 19 | 0.047 | -36 | 2 | 14 | 4.02 |
| 0.993 | 16 | 0.066 | 16 | -54 | 80 | 3.86 |
| - | - | - | 12 | -60 | 76 | 3.55 |
| 1.000 | 7 | 0.209 | 60 | 24 | 10 | 3.74 |
| 0.999 | 13 | 0.094 | -24 | -38 | -32 | 3.60 |
| 1.000 | 5 | 0.286 | -28 | -18 | 80 | 3.56 |
| 1.000 | 6 | 0.243 | 58 | 10 | 30 | 3.53 |
| 1.000 | 6 | 0.243 | 54 | -30 | -0 | 3.52 |
| 1.000 | 5 | 0.286 | 22 | -62 | 48 | 3.50 |

*Note.* Brain regions were identified via AAL atlas. All coordinates were presented in Montreal Neurological Institute (MNI) space. Cluster threshold k >5 voxels, *p* < 0.001.

- - 1. ***Receipts of Monetary Wins vs. Receipts of Missed Monetary Wins***

There were no significant group differences after multiple comparisons at a whole-brain level while receiving *monetary wins vs. receiving missed monetary* wins (cluster threshold k >5 voxels, p < 0.001; see Figure S5).


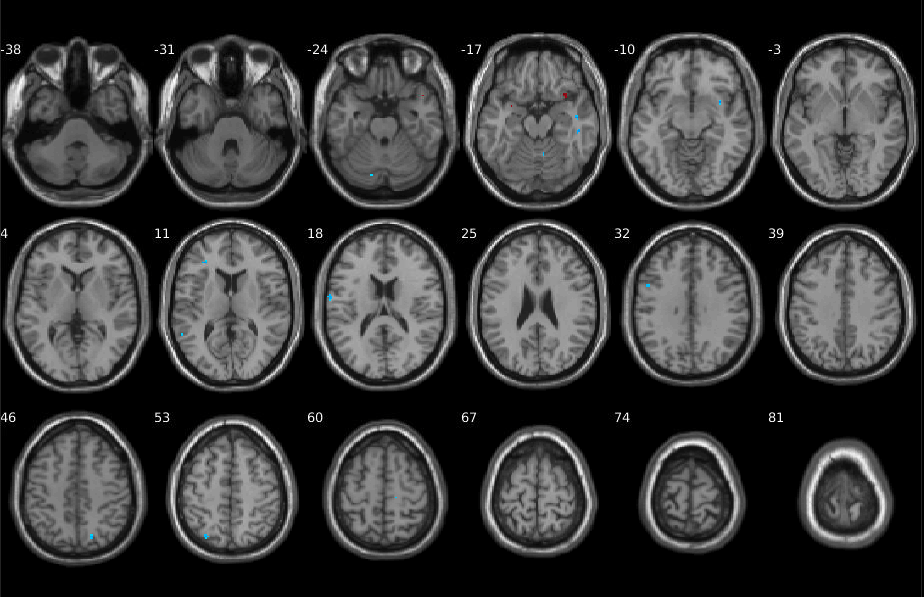
**Figure S5.** *Overview of Brain Activity in CUD Compared to Controls while Receiving Monetary Wins > Receiving Missed Wins*

Note. Cluster threshold k >5 voxels, *p* < 0.001.

| Cluster-level | | | MNI Coordinates | | | T score |
| --- | --- | --- | --- | --- | --- | --- |
| *P*  _FWE-corr_ | *k _E_* | *P*  _uncorrected_ | X | Y | Z |  |
| *receipts of wins > missed wins* | | | | | | |
| 0.978 | 15 | 0.117 | 48 | 16 | -26 | 3.92 |
| 0.998 | 9 | 0.292 | 34 | 16 | -16 | 3.72 |
| 0.999 | 8 | 0.321 | -30 | 4 | 18 | 3.56 |
| 0.942 | 19 | 0.132 | -28 | -66 | 52 | -3.19 |
| 1.000 | 6 | 0.391 | -14 | -78 | -24 | -3.19 |
| 1.000 | 6 | 0.391 | 50 | -4 | 24 | -3.19 |
| 0.995 | 11 | 0.245 | 20 | -68 | 46 | -3.19 |
| 1.000 | 7 | 0.353 | 8 | -52 | -20 | -3.20 |
| 0.917 | 21 | 0.115 | -62 | -8 | 18 | -3.20 |
| 0.963 | 17 | 0.153 | -30 | 34 | 8 | -3.20 |
| 0.992 | 12 | 0.225 | 32 | 22 | 24 | -3.21 |
| 1.00 | 5 | 0.435 | 54 | -42 | 16 | -3.21 |
| 0.997 | 19 | 0.267 | 36 | 10 | -10 | -3.21 |
| 0.978 | 15 | 0.177 | -46 | 10 | 30 | -3.21 |
| 0.997 | 10 | 0.267 | -58 | -50 | 8 | -3.22 |
| 1.000 | 6 | 0.391 | 50 | -24 | -18 | -3.22 |
| 1.000 | 5 | 0.435 | 42 | -14 | 58 | -3.24 |
| 1.000 | 5 | 0.435 | 14 | -24 | 58 | -3.26 |
| 0.998 | 9 | 0.292 | 16 | -12 | -14 | -3.29 |

**Table S3.** *Overview of Location and Strength of Peak Clusters Showing Non-significant Brain Activity in CUD compared to Controls while Receiving Monetary wins > Receiving Missed Wins*

*Note.* Brain regions were identified via AAL atlas. All coordinates were presented in Montreal Neurological Institute (MNI) space. Cluster threshold k >5 voxels, *p* < 0.001.

- 1. **Neuroimaging Results from ROI Analysis**
     1. ***ROI Beta Values***

Table S4 summarises the mean ROI beta values in CUD vs. controls across the three MID contrasts: (1) receiving monetary wins > neutral wins, (2) receiving monetary wins > receiving missed wins*,* and (3) anticipating monetary cues > neutral cues.

**Table S4:** Residual Values within Each ROI, after Controlling for Age and Sex as Covariates

|  | **CUD** | | **HC** | | **Group Difference** | | | **Effect Size** |
| --- | --- | --- | --- | --- | --- | --- | --- | --- |
|  | ***M* (*SD*)** | **Min-Max** | ***M* (*SD*)** | **Min-Max** | ***Z*** | ***p*** | ***95% CI*** | ***d*** |
| ***Receiving Monetary Wins > Neutral Wins*** |  |  |  |  |  |  |  |  |
| Insula, *right* | .07 (1.00) | -2.95-2.20 | -.38 (.92 | -2.13-2.03 | -2.52 | **.012*** | -0.86 to -0.04 | 0.47 |
| Insula, *left* | .08 (.91) | -2.01-2.11 | -.35 (1.06) | -2.72-1.95 | -1.98 | **.047*** | -0.85 to -0.02 | 0.44 |
| Putamen, *right* | .14 (1.03) | -2.33-3.13 | -.27 (.87) | -2.26-1.52 | -2.08 | **.038*** | -0.81 to -0.01 | 0.43 |
| Putamen, *left* | .03 (1.09) | -4.48-2.17 | -.14 (.80) | -1.88-1.51 | -1.27 | .205 | -0.59 to 0.25 | 0.18 |
| Ventral striatum, *right* | .50 (1.14) | -2.80-3.12 | .27 (.67) | -1.09-1.65 | -1.56 | .120 | -0.89 to 0.08 | 0.25 |
| Ventral striatum, *left* | .43 (1.03) | -1.77-2.90 | .30 (.93) | -1.78-1.88 | -.53 | .598 | -0.55 to 0.30 | 0.13 |
| Dorsal caudate, *right* | .19 (1.14) | -3.11-2.80 | -.04 (.67) | -1.40-1.33 | -1.56 | .120 | -0.65 to 0.19 | 0.25 |
| Dorsal caudate, *left* | .38 (1.11) | -2.67-3.29 | .10 (.72) | -1.33-1.39 | -1.64 | .100 | -0.71 to 0.13 | 0.30 |
| Cingulate cortex | .26 (1.02) | -2.78-2.92 | .11 (.95) | -2.76-1.67 | -.38 | .702 | -0.57 to 0.27 | 0.15 |
| Orbitofrontal cortex | .25 (.86) | -3.36-1.82 | .12 (1.18) | -2.34-3.48 | -1.14 | .255 | -0.55 to 0.29 | 0.13 |
| ***Receiving Monetary Wins vs. Missed Wins*** |  |  |  |  |  |  |  |  |
| Insula, *right* | -.26 (1.16) | -5.11-1.75 | -.41 (.63) | -1.95-.68 | -1.80 | .071 | -0.58 to 0.27 | 0.16 |
| Insula, *left* | -.12 (1.10) | -3.19-2.77 | -.22 (.79) | -1.77-1.26 | -.69 | .491 | -0.53 to 0.33 | 0.10 |
| Putamen, *right* | .19 (1.16) | -3.22-2.74 | -.08 (.62) | -1.40-1.54 | -2.08 | **.037*** | -0.69 to 0.16 | 0.29 |
| Putamen, *left* | .43 (1.11) | -2.36-2.81 | .28 (.78) | -1.44-2.16 | -1.14 | .254 | -0.58 to 0.28 | 0.16 |
| Ventral striatum, *right* | .66 (1.11) | -4.38-3.54 | .46 (.75) | -1.62-2.20 | -1.28 | .201 | -0.63 to 0.22 | 0.21 |
| Ventral striatum, *left* | .63 (1.09) | -1.52-3.75 | .52 (.81) | -.98-3.06 | -.65 | .517 | -0.54 to 0.31 | 0.11 |
| Dorsal caudate, *right* | .37 (1.16) | -2.50-2.74 | .19 (.63) | -1.26-1.93 | -1.35 | .176 | -0.61 to 0.24 | 0.19 |
| Dorsal caudate, *left* | .54 (1.15) | -2.40-3.92 | .37 (.64) | -1.09-1.68 | -.77 | .441 | -0.60 to 0.26 | 0.18 |
| Cingulate cortex | -.12 (1.08) | -3.45-1.88 | .08 (.82) | -1.78-2.00 | -.43 | .670 | -0.23 to 0.63 | -0.21 |
| Orbitofrontal cortex | .21 (.97) | -2.22-2.27 | .35 (1.04) | -1.19-3.74 | -.21 | .831 | -0.29 to 0.57 | -0.14 |
| ***Anticipating Monetary Cues vs. Neutral Cues*** |  |  |  |  |  |  |  |  |
| Insula, *right* | .06 (1.05) | -2.24-4.28 | -.09 (.89) | -1.77-1.91 | -.74 | .462 | -0.57 to 0.30 | 0.15 |
| Insula, *left* | .04 (1.02) | -2.11-4.74 | -.04 (.96) | -1.74-2.11 | -.28 | .781 | -0.50 to 0.35 | 0.08 |
| Putamen, *right* | .05 (1.10) | -2.24-3.79 | -.03 (.79) | -1.27-2.02 | -.31 | .757 | -0.51 to 0.34 | 0.08 |
| Putamen, *left* | .05 (1.02) | -2.17-3.89 | .01 (.94) | -1.50-2.79 | .00 | .997 | -0.46 to 0.39 | 0.04 |
| Ventral striatum, *right* | .02 (1.11) | -4.20-2.59 | -.07 (.77) | -1.80-1.26 | -.62 | .538 | -0.51 to 0.33 | 0.09 |
| Ventral striatum, *left* | .13 (1.05) | -1.97-2.79 | -.21 (.85) | -2.43-1.52 | -1.60 | .109 | -0.75 to 0.09 | 0.36 |
| Dorsal caudate, *right* | .02 (1.12) | -2.57-3.47 | -.11 (.75) | -1.62-1.75 | -.44 | .661 | -0.55 to 0.29 | 0.14 |
| Dorsal caudate, *left* | -.03 (1.13) | -2.70-3.11 | -.03 (.71) | -1.41-1.52 | -.06 | .952 | -0.42 to 0.42 | 0.94 |
| Cingulate cortex | .01 (1.06) | -2.30-2.52 | .04 (.88) | -2.42-1.61 | -.38 | .702 | -0.40 to 0.45 | -0.03 |
| Orbitofrontal cortex | .08 (.98) | -2.10-2.74 | -.06 (1.02) | -2.64-2.13 | -.38 | .702 | -0.57 to 0.28 | 0.14 |

Note. CUD = Cannabis Use Disorder; *M* = mean; *SD* = standard deviation; Z = nonparametric Mann-Whitney *U* test; CI = Confidence Intervals; d = Cohen’s d; Effect size interpretation: < 0.30 = small; 0.30 – 0.49 = moderate; ≥ 0.50 = large; ****p* < .05.**

- - 1. ***Anticipating Monetary Cues vs. Anticipating Neutral Cues***

There were no significant group differences in the activity of any ROIs while anticipating monetary cues vs. neutral cues (see Figure S6).

**Figure S6.** *Overview of Results from ROI Analyses, Showing ROI Activity (Beta Values Adjusted for Age and Sex on the Y-axis, Groups on the X-axis: Arbitrary Unit) in CUD vs. Controls while Anticipating Monetary Cues vs. Neutral Cues*


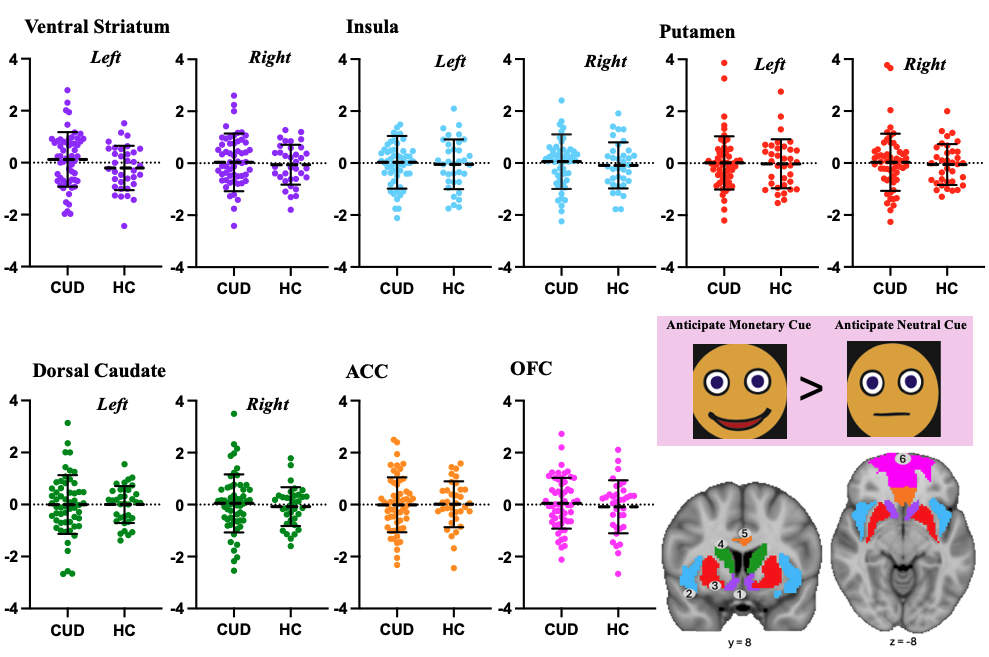


Note. CUD = Cannabis Use Disorder; HC = Healthy Control; ACC = Anterior Cingulate Cortex; OFC = Orbitofrontal Cortex; brain image colours signify the selected ROIs: (1) ventral striatum; (2) insula; (3) putamen; (4) dorsal caudate; (5) cingulate cortex; (6) orbitofrontal cortex; adapted from [19]; blacklines represent 95% error bar, indicating a 5% likelihood that the value falls outside the error span.

- 1. **Brain-Behaviour Correlations**

As shown in Figure S7, in the CUD group, there were significant correlations without controlling for multiple comparisons between greater task-based activity and AES scores, as well as the age of regular cannabis use. Specifically, for the contrast *receiving* *monetary wins vs. neutral wins,* greater activity of the left insula and right putamen significantly correlated with a later age of onset of regular use, while greater activity of the bilateral insula and right putamen correlated with greater apathy levels (AES scores). The other ROIs did not show significant correlations for this contrast. In addition, greater activity of the right putamen when receiving *monetary wins vs.* *missed wins* correlated with greater apathy levels (see Figure S7). The other ROIs did not show significant correlations for this contrast.

*
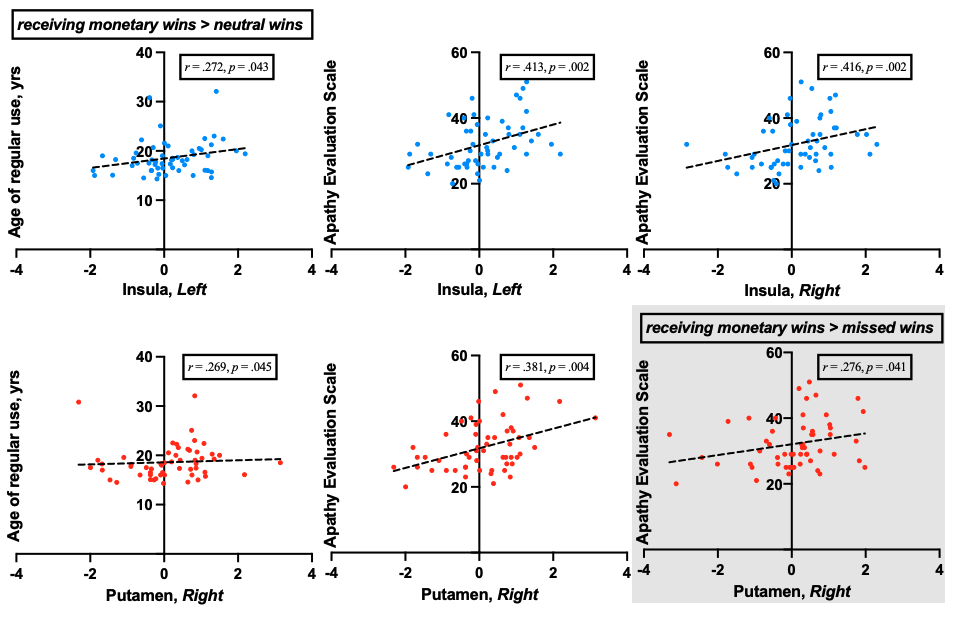
***Figure S7.** *Overview of Significant Correlations for the CUD Group, Between the ROIs Insula and Putamen, and the Age of Cannabis Use Onset, AES Scores During the Receipt of Monetary Wins, and Between Putamen Activity and AES Scores while Receiving Monetary Wins > Missed Wins.*

Note. Yrs = years; the white box represents significant ROI correlations while *receiving monetary wins > neutral wins*; the grey box represents significant ROI correlations while *receiving monetary wins > missed wins.*

- 1. **Additional Whole-Brain Analysis Signifying the Activation Patterns for Each Group**
     1. ***Anticipation of Monetary Cues vs. Anticipation of Neutral Cues***

**Figure S8.** *Overview of Pattern Activation in the CUD Group while Anticipating Monetary Cues > Anticipating Neutral Cues*

*
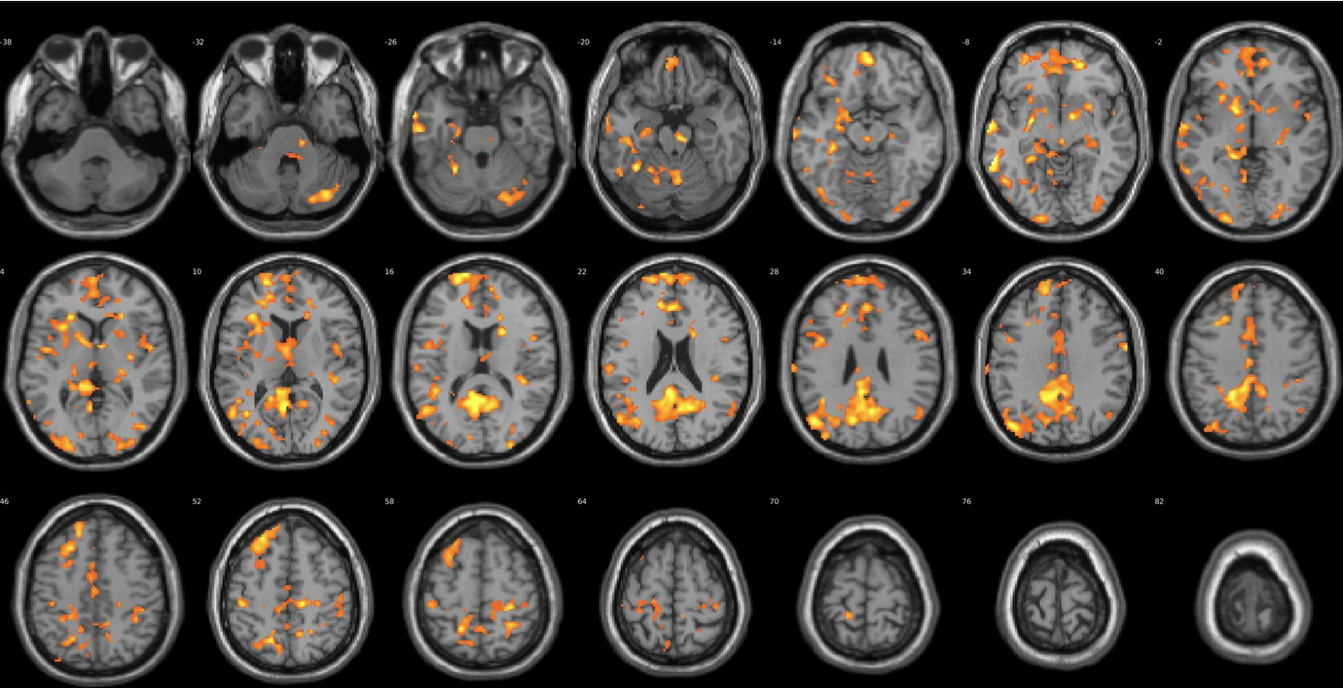
*

Note. Cluster threshold k >50 voxels, *p* < 0.05.

**Figure S9.** *Overview of Pattern Activation in the Control Group while Anticipating Monetary Cues > Anticipating Neutral Cues*


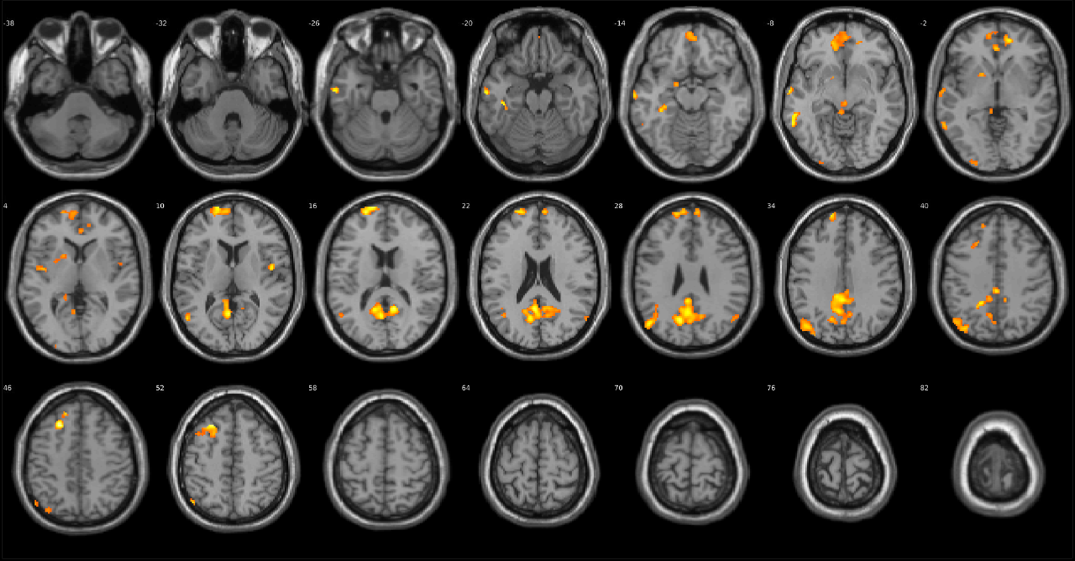


Note. Cluster threshold k >50 voxels, *p* < 0.05.

- - 1. ***Receipts of Monetary Wins vs. Receipt of Neutral Wins***

**Figure S10.** *Overview of Pattern Activation in the CUD Group while Receiving Monetary Wins> Receiving Neutral Wins*

*
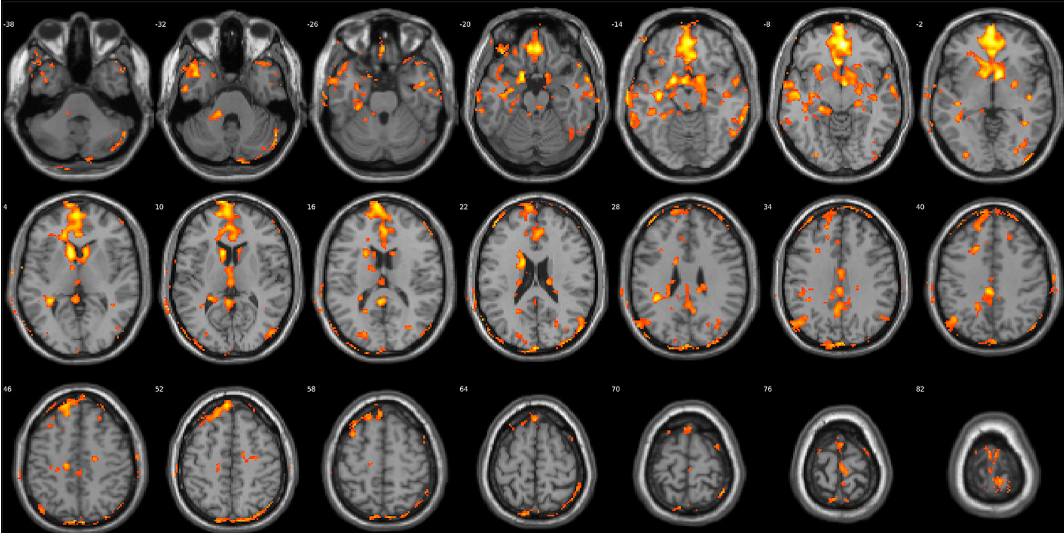
*

Note. Cluster threshold k >50 voxels, *p* < 0.05.

**Figure S11.** *Overview of Pattern Activation in the Control Group while Receiving Monetary Wins> Receiving Neutral Wins*


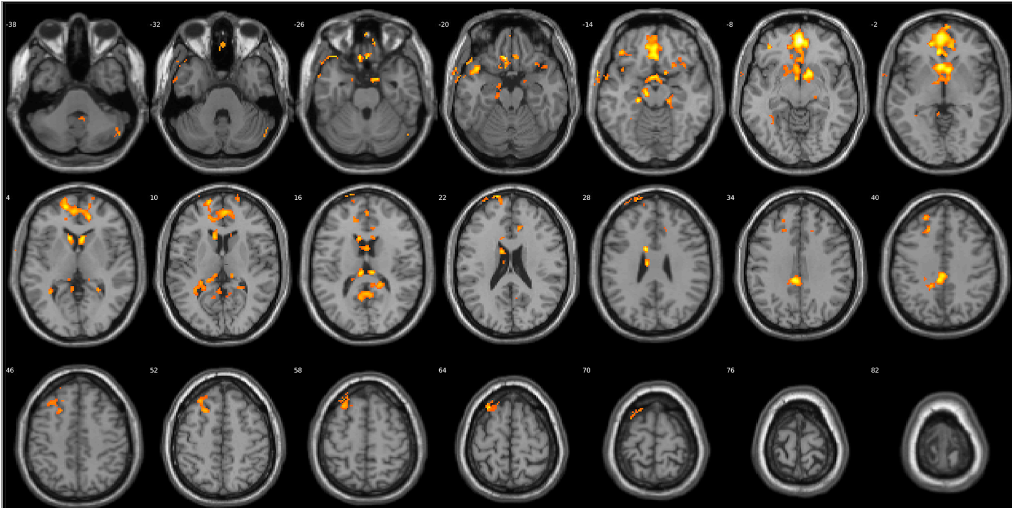


Note. Cluster threshold k >50 voxels, *p* < 0.05.

- - 1. ***Receipts of Monetary Wins vs. Receipt of Missed Monetary Wins***

**Figure S12.** *Overview of Pattern Activation in the CUD Group while Receiving Monetary Wins> Receiving Missed Wins*

*
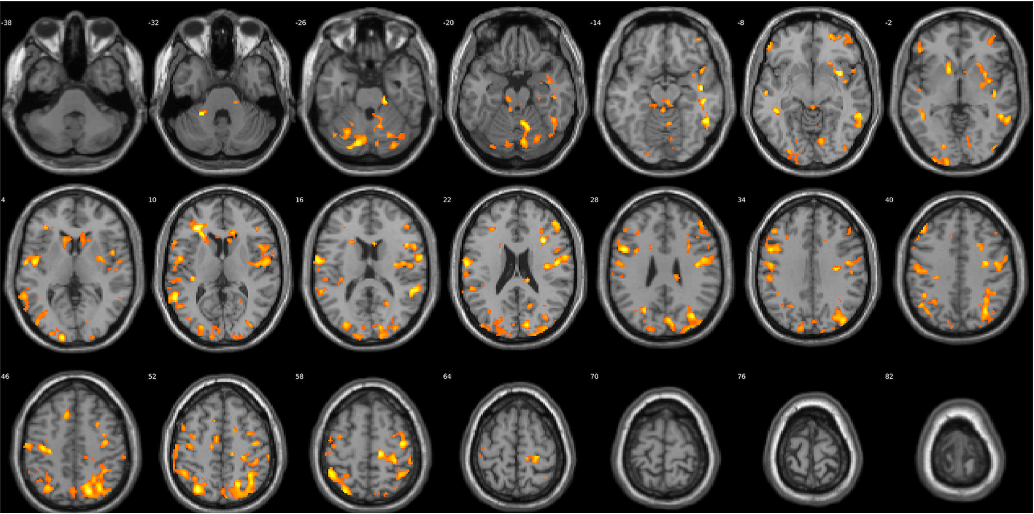
*

Note. Cluster threshold k >50 voxels, *p* < 0.05.

**Figure S13.** *Overview of Pattern Activation in the Control Group while Receiving Monetary Wins> Receiving Missed Wins*

*
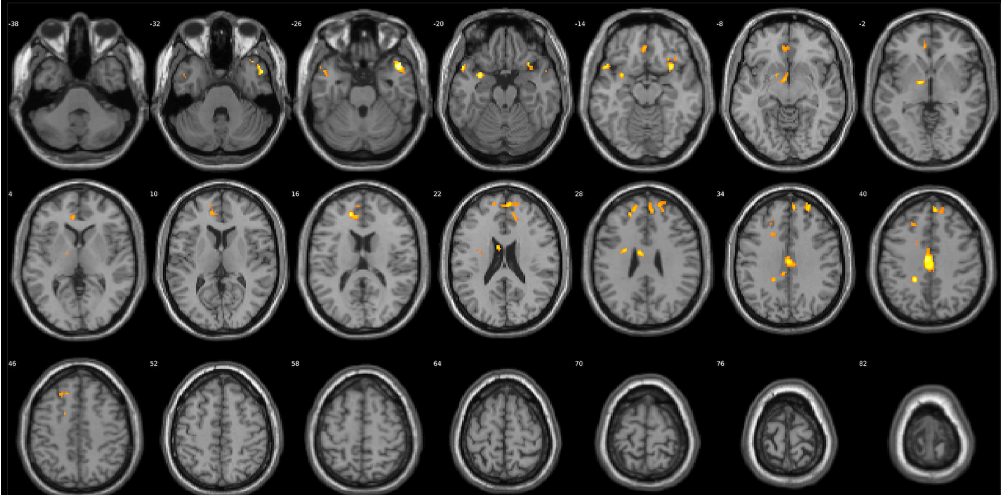
*

Note. Cluster threshold k >50 voxels, *p* < 0.05.

**References**

1. First MB, Williams JB, Karg RS, Spitzer RL. SCID-5-RV. 2015.

2. Sheehan D, Lecrubier Y, Janavs J. International Neuropsychiatric Interview (MINI). Tampa, FL: University of South Florida Institute for Research in Psychiatry …; 1994.

3. Babor TF, Higgins-Biddle JC, Saunders JB, Monteiro MG, Organization WH. AUDIT: the alcohol use disorders identification test: guidelines for use in primary health care. World Health Organization, 2001.

4. Wechsler D. Wechsler abbreviated scale of intelligence. 1999.

5. Lovibond PF, Lovibond SH. The structure of negative emotional states: Comparison of the Depression Anxiety Stress Scales (DASS) with the Beck Depression and Anxiety Inventories. *Behaviour Research and Therapy*. 1995;33(3):335-43.

6. Veale JF. Edinburgh Handedness Inventory – Short Form: A revised version based on confirmatory factor analysis. *Laterality*. 2014;19(2):164-77.

7. Lorenzetti V, Solowij N, Whittle S, Fornito A, Lubman DI, Pantelis C, et al. Gross morphological brain changes with chronic, heavy cannabis use. *The British Journal of Psychiatry*. 2015;206(1):77-8.

8. Solowij N, Stephens RS, Roffman RA, Babor T, Kadden R, Miller M, et al. Cognitive functioning of long-term heavy cannabis users seeking treatment. *JAMA*. 2002;287(9):1123-31.

9. Yucel M, Solowij N, Respondek C, Whittle S, Fornito A, Pantelis C, et al. Regional brain abnormalities associated with long-term heavy cannabis use. *Arch Gen Psychiatry*. 2008;65(6):694-701.

10. Allsop DJ, Norberg MM, Copeland J, Fu S, Budney AJ. The Cannabis Withdrawal Scale development: patterns and predictors of cannabis withdrawal and distress. *Drug Alcohol Depend*. 2011;119(1-2):123-9.

11. Sobell LC, Sobell MB. Timeline follow-back: A technique for assessing self-reported alcohol consumption. Measuring alcohol consumption: Psychosocial and biochemical methods: Springer; 1992. p. 41-72.

12. Heatherton TF, Kozlowski LT, Frecker RC, Fagerstrom KO. The Fagerstrom Test for Nicotine Dependence: a revision of the Fagerstrom Tolerance Questionnaire. *Br J Addict*. 1991;86(9):1119-27.

13. Beck AT, Steer RA, Brown GK. Beck depression inventory. 1996.

14. Konings M, Bak M, Hanssen M, van Os J, Krabbendam L. Validity and reliability of the CAPE: a self-report instrument for the measurement of psychotic experiences in the general population. *Acta Psychiatr Scand*. 2006;114(1):55-61.

15. Cohen S, Kamarck T, Mermelstein R. A global measure of perceived stress. *J Health Soc Behav*. 1983;24(4):385-96.

16. Spielberger C. State-trait anxiety inventory for adults. *Mind Garden*. 1983.

17. Marin RS, Biedrzycki RC, Firinciogullari S. Reliability and validity of the Apathy Evaluation Scale. *Psychiatry Res*. 1991;38(2):143-62.

18. Knutson B, Adams CM, Fong GW, Hommer D. Anticipation of increasing monetary reward selectively recruits nucleus accumbens. *The Journal of neuroscience*. 2001;21(16):RC159.

19. Hoogendam JM, Kahn RS, Hillegers MH, van Buuren M, Vink M. Different developmental trajectories for anticipation and receipt of reward during adolescence. *Dev Cogn Neurosci*. 2013;6:113-24.

20. Parkes L, Fulcher B, Yucel M, Fornito A. An evaluation of the efficacy, reliability, and sensitivity of motion correction strategies for resting-state functional MRI. *Neuroimage*. 2018;171:415-36.

21. Tukey JW. Exploratory data analysis: Springer; 1977.
